# Interaction of transcription factor AP‐2 gamma with proto‐oncogene PELP1 promotes tumorigenesis by enhancing RET signaling

**DOI:** 10.1002/1878-0261.12871

**Published:** 2021-02-09

**Authors:** Junhao Liu, Zexuan Liu, Mengxing Li, Weiwei Tang, Uday P. Pratap, Yiliao Luo, Kristin A. Altwegg, Xiaonan Li, Yi Zou, Hong Zhu, Gangadhara R. Sareddy, Suryavathi Viswanadhapalli, Ratna K. Vadlamudi

**Affiliations:** ^1^ UT Health San Antonio Long School of Medicine Department of Obstetrics and Gynecology UT Health San Antonio TX USA; ^2^ Department of Oncology Xiangya Hospital Central South University Hunan China; ^3^ Department of Respiratory Medicine Xiangya Hospital Central South University Hunan China; ^4^ Department of Obstetrics and Gynecology Affiliated Hospital of Integrated Traditional Chinese and Western Medicine Nanjing University of Chinese Medicine China; ^5^ Department of General Surgery Xiangya Hospital Central South University Hunan China; ^6^ UT Health San Antonio Mays Cancer Center‐ MD Anderson Cancer Center UT Health San Antonio TX USA; ^7^ Greehey Children's Cancer Research Institute UT Health San Antonio TX USA

**Keywords:** breast cancer, coactivator, PELP1, TFAP2C, therapy resistance

## Abstract

A significant proportion of estrogen receptor‐positive (ER+) breast cancer (BC) initially responds to endocrine therapy but eventually evolves into therapy‐resistant BC. Transcription factor AP‐2 gamma (TFAP2C) is a known regulator of ER activity, and high expression of TFAP2C is associated with a decreased response to endocrine therapies. PELP1 is a nuclear receptor coregulator, commonly overexpressed in BC, and its levels are correlated with poorer survival. In this study, we identified PELP1 as a novel interacting protein of TFAP2C. RNA‐seq analysis of PELP1 knockdown BC cells followed by transcription factor motif prediction pointed to TFAP2C being enriched in PELP1‐regulated genes. Gene set enrichment analysis (GSEA) revealed that the TFAP2C‐PELP1 axis induced a subset of common genes. Reporter gene assays confirmed PELP1 functions as a coactivator of TFAP2C. Mechanistic studies showed that PELP1‐mediated changes in histone methylation contributed to increased expression of the TFAP2C target gene *RET*. Furthermore, the TFAP2C‐PELP1 axis promoted the activation of the RET signaling pathway, which contributed to downstream activation of AKT and ERK pathways in ER+ BC cells. Concomitantly, knockdown of PELP1 attenuated these effects mediated by TFAP2C. Overexpression of TFAP2C contributed to increased cell proliferation and therapy resistance in ER+ BC models, while knockdown of PELP1 mitigated these effects. Utilizing ZR75‐TFAP2C xenografts with or without PELP1 knockdown, we provided genetic evidence that endogenous PELP1 is essential for TFAP2C‐driven BC progression *in vivo*. Collectively, our studies demonstrated that PELP1 plays a critical role in TFAP2C transcriptional and tumorigenic functions in BC and blocking the PELP1‐TFAP2C axis could have utility for treating therapy resistance.

## Graphical abstract

Transcription factor AP‐2 gamma (TFAP2C) is a known regulator of the estrogen receptor, and high expression of TFAP2C is associated with therapy resistance. This study identified PELP1 as a TFAP2C interacting protein. PELP1 is essential for optimal TFAP2C transcriptional functions. TFAP2C interactions with PELP1 confer a growth advantage to breast cancer (BC) cells by activating an oncogenic RET signaling thus contributing to BC progression and therapy resistance.

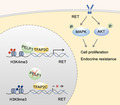

**Abbreviations**
ATCC: American‐Type Culture Collection
BC: breast cancer
ER+: estrogen receptor positive
GSEA: gene set enrichment analysis
IP: immunoprecipitation
NRs: nuclear receptors
PELP1: Proline‐, glutamic acid‐, and leucine‐rich protein 1
TFAP2C: transcription factor AP‐2 gamma
TFs: transcription factors

## 1 Introduction

Breast cancer (BC) is the most common malignancy in women, the second leading cause of cancer‐related death after lung cancer . The majority of BC (70%) is estrogen receptor positive (ER+). Therapies for ER+ BC involve modulation of ER signaling using either antiestrogens or aromatase inhibitors. However, many of ER+ BC patients will eventually acquire resistance to these drugs, and disease progression is common. The activation of additional nuclear receptors (NRs) and transcription factors (TFs) is suspected to contribute to the progression of ER+ BC under the current standard endocrine therapy treatment. At this time, little is known about the mechanisms by which alternative oncogenic signaling pathways contribute to ER+ BC progression.

Transcription factor AP‐2 gamma (TFAP2C) is a member of the AP‐2 family, which functions as a sequence‐specific TF and activates a number of developmental genes in multiple organs [1]. TFAP2C plays an important role in the development of the mammary gland [2], regulation of luminal specific genes [3], hormone responsive BC progression [4], and progression of HER2‐amplified BC [5]. TFAP2C is an independent predictor of poor survival and higher levels of TFAP2C contribute to endocrine therapy resistance [6]. However, the molecular mechanism(s) of TFAP2C‐mediated BC progression are not completely understood.

Proline‐, glutamic acid‐, and leucine‐rich protein 1 (PELP1) [7], originally cloned in our lab, plays a critical role in multiple NR signaling resulting in BC progression [8, 9]. PELP1 has an essential role in several pathways including hormonal signaling, cell cycle progression, ribosomal biogenesis, and DNA damage response [9, 10, 11]. Notably, PELP1 protein has a histone‐binding domain [12], recognizes histone modifications, and interacts with several chromatin‐modifying complexes including: KDM1A [12], HDAC [13], PRMT [14], and CARM1 [15]. PELP1 is a known prognostic indicator of poorer BC survival [16], and its dysregulation contributes to BC therapy resistance [17, 18] and metastases [13]. In addition, dysregulation of PELP1 expression in BC [19] promotes epigenetic alterations [12]. However, the mechanisms by which PELP1 promotes therapy resistance remain elusive.

In this study, we demonstrate that PELP1 interacts with and functions as a coactivator of TFAP2C. Further, we show that knockdown of PELP1 attenuates TFAP2C‐mediated oncogenic functions both *in vitro* and *in vivo*. Mechanistic studies show that PELP1 knockdown promotes inhibitory histone methylation marks at TFAP2C‐binding sites at the target gene promoter. Accordingly, PELP1 participates in the activation of a subset of TFAP2C genes and plays an integral role in TFAP2C‐mediated activation of RET signaling. This observation is functionally significant as increased expression of both PELP1 and TFAP2C occurs during BC progression and both are implicated in the development of endocrine therapy resistance.

## 2 Materials and methods

### 2.1 Cell cultures and reagents

MCF7 and ZR75 cell lines were purchased from the American‐Type Culture Collection (ATCC, Manassas, Virginia) and maintained in RPMI 1640 medium supplemented with 10% FBS (Millipore Sigma, St. Louis, MO, USA). HEK293T cell line was purchased from ATCC and maintained in Dulbecco's Modified Eagle's medium (DMEM) supplemented with 10% FBS. For Fulvestrant (MedChemExpress, Monmouth Junction, NJ, USA) treatment, both MCF7 and ZR75 cells were hormone stripped in phenol red‐free media with 5% dextran‐coated charcoal‐treated FBS (Gemini Bio Products, West Sacramento, CA, USA). After 2 days of incubation, cells were seeded into six‐well plates and treated with Fulvestrant in the presence of 10 nm 17‐β‐estradiol (E2; Sigma). All model cells utilized were free of mycoplasma contamination, and STR DNA profiling of the cells was used to confirm identity. The TFAP2C (sc‐12762) and GFRA1 (sc‐271546) antibodies were purchased from Santa Cruz Biotechnology (Dallas, TX, USA). The PELP1 antibody (A300‐180A) was purchased from Bethyl Laboratories (Montgomery, TX, USA). The ERα (04‐820) antibody was purchased from Sigma. The p‐ERK1/2 (9106), ERK1/2 (9102), p‐Akt (9271), Akt (9272), RET (3223), GST (2624), and GAPDH (8884) antibodies were obtained from Cell Signaling Technology (Beverly, MA, USA). The Ki67 (ab16667) antibody was purchased from Abcam (Cambridge, MA, USA).

### 2.2 Generation of TFAP2C model cell lines

MCF7 and ZR75 cells stably expressing TFAP2C were generated using EF1a_TFAP2C_P2A_Hygro (a gift from Prashant Mali, Addgene plasmid # 120487, Watertown, MA). MCF7 and ZR75 cells stably expressing TFAP2C‐shRNA cells were generated using TFAP2C MISSION shRNA particles (TRCN0000019746; Sigma, Indianapolis, IN, USA). MCF7, ZR75, and HEK293T cells stably expressing PELP1‐shRNA were generated using validated human‐specific lentiviral PELP1‐shRNA particles (TRCN0000159883, TRCN0000159673; Sigma). MCF7 and ZR75 cells stably expressing RET‐shRNA were generated using validated human‐specific lentiviral RET‐shRNA (TRCN0000195426, TRCN0000194736; Sigma). MCF7 and ZR75 cells stably expressing PELP1‐GFP were described previously [20]. Lentiviral particles expressing nontargeted shRNA (SHC016; Sigma) were used to generate control cells. Stable clones were selected with puromycin (1 μg·mL^−1^) or hygromycin (100 μg·mL^−1^). Pooled clones were used for all subsequent studies.

### 2.3 GST pull down, immunoprecipitation, and western blot assays

Whole cell lysates were prepared using RIPA buffer, and western blot analysis was performed using phospho‐specific antibodies as described [21]. For the RET signaling, MCF7 and ZR75 cells were serum‐starved for 24 h and then changed to 10% FBS normal medium for 30 min. The RET inhibitor, BLU667, was purchased from ChemieTek (Indianapolis, IN, USA). For immunoprecipitation (IP), the lysates were precleared with protein A beads followed by TFAP2C antibody or GFP‐TRAP beads (ChromoTek, Planegg‐Martinsried, Germany). Construction of GST full‐length PELP1 [15] and GST‐PELP1 deletions [22] have been described previously. For GST pull‐down assays, lysates were incubated with various PELP1‐GST fragments, bound proteins were isolated by GST pull‐down assay as previously described [22], and the interaction of PELP1 with TFAP2C was analyzed by western blot analysis. Mouse True Blot HRP secondary antibody (cat#18‐8817‐31, eBioscience, Thermo Fisher Scientific, Waltham, MA, USA) was used in the TFAP2C IP.

### 2.4 AP2 reporter gene assays

The pGL3‐AP2‐LUC plasmid was a kind gift from Dawid Igor [23]. MCF7, ZR75, and HEK293T model cell lines were seeded into 24‐well plates and after overnight incubation, transiently transfected with 250 ng AP2‐LUC reporter vector along with PELP1 shRNA vector (SuperArray Bioscience Corporation, Frederick, MD, USA), TFAP2C (GFP‐tagged) expressing vector (RG208665; OriGene, Rockville, MD, USA) or control vector using Turbofect Transfection Reagent (Thermo Fisher Scientific). Renilla reporter (50 ng) plasmid was co‐transfected and used for normalization of transfection efficiency. Cells were lysed in luciferase lysis buffer, and the luciferase activity was measured using the Dual Luciferase Assay System (Promega, Madison, WI, USA) with a luminometer.

### 2.5 Cell viability, colony formation, and soft agar assays

The cell viability of the BC model cells expressing TFAP2C or control shRNA was assessed by using MTT assay as described [24]. For colony formation assays, BC model cells (500 cells per well) were seeded into six‐well plates and are allowed to grow for 14 days as described [24]. The cells were fixed in ice‐cold methanol and stained with 0.5% crystal violet solution. Soft agar colony‐growth assays were done as previously described [19]. Briefly, 1 mL of 0.6% Difco Bactro Agar in RPMI 1640 medium was added to the bottom of the plate. BC model cells (1 × 10^4^) mixed with 1 mL of 0.36% agar solution in RPMI were layered on top of the 0.6% agar layer. The colonies were stained and counted after 21 days. Colony counting experiments were blinded and independently scored by two coauthors.

### 2.6 Gene set enrichment analysis

The PELP1 RNA‐seq data used in this study were obtained from ZR75 cells and were previously published [14]. TFAP2C‐induced gene set and TFAP2C‐repressed gene set were defined according to TFAP2C microarray data (GSE44203) [3] by *P* value < 0.01 and |log_2_ fold change| > 1. GSEA (Broad Institute, http://www.broadinstitute.org/gsea/index.jsp) was performed using C3: TF targets gene sets in the MSigDB database or customized TFAP2C gene set.

### 2.7 ChIP assays

ChIP assay was performed using Pierce™ Magnetic ChIP Kit (Thermo Fisher) according to the manufacturer's protocol. Briefly, 6 × 10^6^ cells were cross‐linked using 1% formaldehyde for 10 min, lysed, and digested with micrococcal nuclease. ChIP was performed using 2.5 μg antibodies of H3K9me3 (ab8898; Abcam), H3K4me3 (C15410003‐50; Diagenode, Denville, NJ, USA), TFAP2C (sc‐12762x ; Santa Cruz), PELP1 (A300‐180A; Bethyl), or 2.5 μg of isotype control IgG antibody. ChIP DNA was resuspended in 50μL TE buffer and used for RT–qPCR amplification using the gene‐specific primers: RET −101.5 kb forward: 5′‐TACTGTCAAGCCACGTCAGC‐3′, reverse: 5′‐CGCTACCTCTAACAGGCGAA‐3′. RET −50.7 kb forward: 5′‐ACGCATTGTTCACCCACTGA‐3', reverse: 5'‐AAGGTTAGAAGCTGCGCTGT‐3′. RET −32.5 kb forward: 5′‐ACTAGCTCCTCACCTACCCG‐3′, reverse: 5′‐CTCTCCTATGCAGGCTGCTC‐3′. RET +5.0 kb forward: 5′‐AAGGACAGCAGTTCAGGTCG‐3′, reverse: 5′‐CACTTTGTGGAGCCACTCCT‐3′. RET −46.8 kb forward: 5′‐AGGGATGGACCAGTCCAAGT‐3′, reverse: 5′‐TCAGAGCCCAAGATGGAGGA‐3′. GAPDH promoter forward: 5′‐CCGGGTCTTTGCAGTCGTAT‐3', reverse: 5'‐GGGAGTAGGGACCTCCTGTT‐3′. For Re‐Chip assay, the first ChIP was performed using TFAP2C antibody, DNA was eluted using 15 mm DTT in TE buffer and was used for secondary ChIP assay with PELP1 or IgG antibody.

### 2.8 RT–qPCR analyses

Total RNA was isolated using TRIzol Reagent (Invitrogen, Carlsbad, CA, USA) according to the manufacturer's instructions. cDNA synthesis was done using the Superscript III RT–PCR kit (Invitrogen). Real‐time PCR was done using gene‐specific primers. Results were normalized to β‐actin transcript levels, and the difference in fold expression was calculated using the delta‐delta‐CT method. Primer sequences used include the following: BMP4 forward: 5′‐ATGATTCCTGGTAACCGAATGC‐3′, reverse: 5′‐CCCCGTCTCAGGTATCAAACT‐3′. GFRA1 forward: 5′‐CCAAGCACAGCTACGGAATG‐3′, reverse: 5′‐CAGGCACGATGGTCTGTCG‐3′. PAK4 forward: 5′‐GGACATCAAGAGCGACTCGAT‐3′, reverse: 5′‐CGACCAGCGACTTCCTTCG‐3′. RET forward: 5′‐ACACGGCTGCATGAGAACAA‐3′, reverse: 5′‐GCCCTCACGAAGGGATGTG‐3′. KRT19 forward: 5′‐AACGGCGAGCTAGAGGTGA‐3′, reverse: 5′‐GGATGGTCGTGTAGTAGTGGC‐3′. TRIAP1 forward: 5′‐CGCTGGTTCGCCGAGAAATTTC‐3′, reverse: 5′‐GCCCATGAACTCCAGTCCTTCA‐3′. TFAP2C forward: 5′‐TCAGTCCCTGGAAGATTGTCG‐3′, reverse: 5′‐CCAGTAACGAGGCATTTAAGCA‐3'. β‐actin forward: 5′‐GTGGGCATGGGTCAGAAG‐3′, reverse: 5′‐TCCATCACGATGCCAGTG‐3′.

### 2.9 Xenograft studies

All animal experiments were performed after obtaining UT Health San Antonio IACUC approval, and all the methods were carried out in accordance with IACUC guidelines. ZR75 cells (2 × 10^6^) were mixed with equal volume of Matrigel ( Corning Inc. Bedford, MA) and implanted in the mammary fat pads of 8‐week‐old SCID mice implanted with an E2 pellet as described previously [13]. Tumor intake was ≂80%, and six mice per group were used in each group. Expect the PI, the investigators conducting tumor measurements were blinded to the study. All analyzed mice were from a single experiment. After tumor establishment, tumor growth was measured with digital caliper at 3–5‐day intervals, and volume was calculated using a modified ellipsoidal formula: tumor volume = 1/2(*L* × *W* ^2^), where *L* is the longitudinal diameter and *W* is the transverse diameter. At the end of the experiment, mice were euthanized, tumors were excised, and processed for histological and biochemical studies.

### 2.10 Immunohistochemistry analyses

Immunohistochemical analysis was performed as described previously [25]. Briefly, tissue sections were blocked with normal horse serum (Vector Labs, Burlingame, CA, USA) followed by overnight incubation with primary antibody [Ki‐67 (1 : 100); RET (1 : 100)] and subsequent secondary antibody incubation for 30 min at room temperature. Percent of Ki‐67‐positive proliferating cells was calculated in five randomly selected microscopic fields. The staining of RET on xenograft tumor slides was quantified using five randomly selected microscopic fields and by using imagej analysis software (NIH, Bethesda, MD, USA). Briefly, the image was subjected to color deconvolution and mean DAB intensity was measured using H DAB vector plug‐in and the resulting D‐HSCORE values were plotted in a histogram [26]. Immunohistochemistry (IHC) experiments were blinded and independently scored by two coauthors.

### 2.11 Statistical analysis

GraphPad Prism 7 software (San Diego, CA, USA) was used to analyze all data. Data represented in bar graphs are shown as mean ± SEM. A Student's *t*‐test was performed for all pairwise comparisons. For multiple experimental groups, statistical data were analyzed using a two‐way ANOVA and Tukey for *post hoc* test. A value of *P* < 0.05 was considered as statistically significant.

## 3 Results

### 3.1 PELP1 interacts with transcription factor TFAP2C

**Fig. 1:**
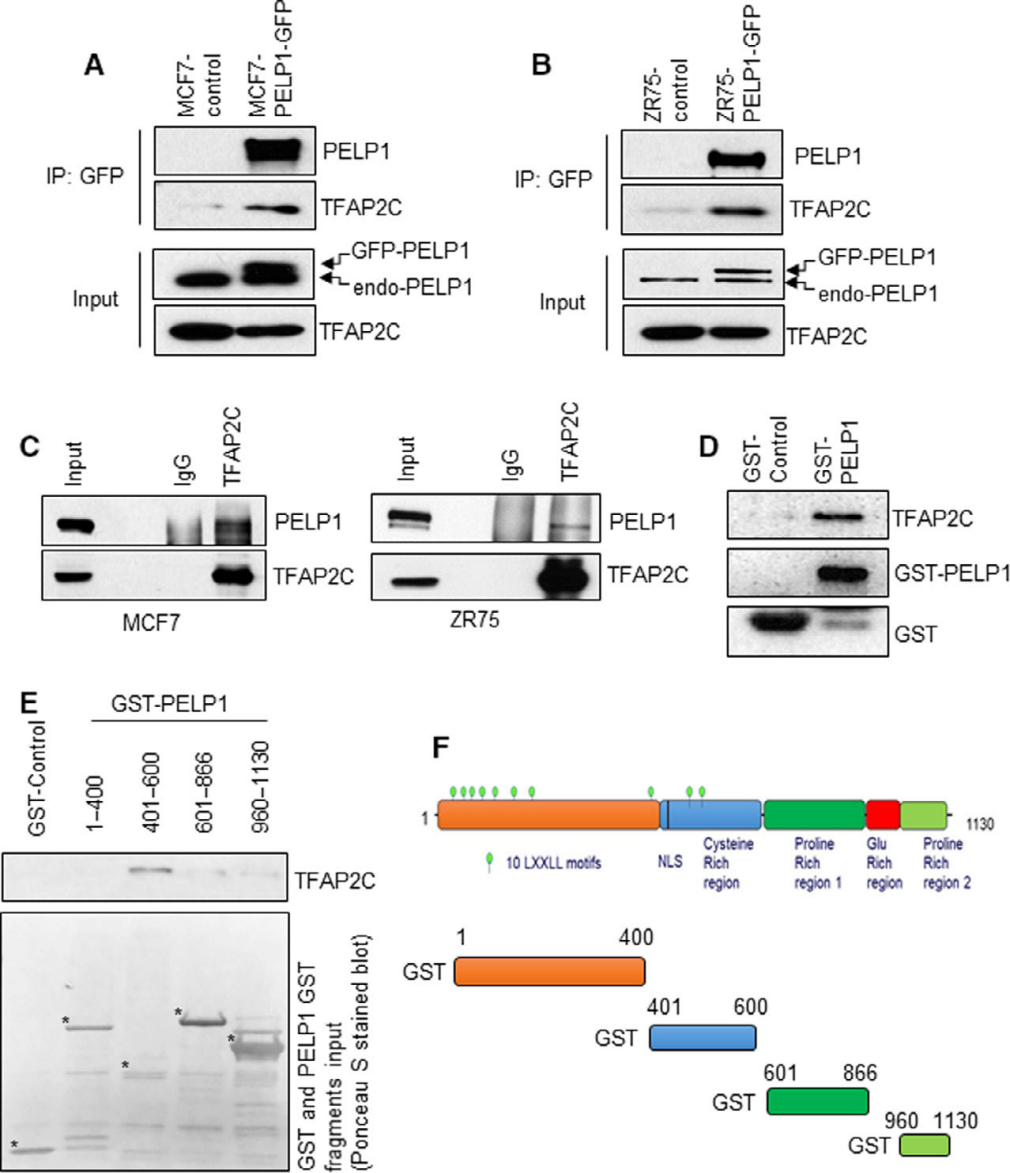
PELP1 interacts with TFAP2C. (A, B) Total lysates from MCF7‐control and MCF7‐GFP‐PELP1 cells (A) or ZR75‐control and ZR75‐GFP‐PELP1 cells (B) were subjected to IP using the GFP‐TRAP beads, and the TFAP2C interaction was verified by western blotting (*n* = 2). (C) Total lysates from MCF7 and ZR75 cells were subjected to IP using the control IgG or TFAP2C antibody, and the PELP1 interaction was verified by western blotting (*n* = 2). (D) Total lysates from MCF7 cells were subjected to GST pull‐down assays using the purified control GST or GST‐PELP1 full‐length proteins expressed in *Escherichia coli* and the TFAP2C interaction was verified by western blotting. (E) *Escherichia coli* expressed and purified GST‐PELP1 deletions were used to identify TFAP2C interacting regions in the PELP1 protein using MCF7 total lysates. (F) Schematic representation of GST‐PELP1 deletions utilized in the pull‐down assays.

Our ongoing yeast‐based library screening identified TFAP2C as a potential PELP1‐binding protein. To verify that the observed interaction between PELP1 and TFAP2C in the yeast screen also occurs in BC cells, we performed IP assays using ER+ BC cell lines. First, we validated the potential interaction between these proteins using GFP epitope tagged PELP1 expressing MCF7 and ZR75 cell lines. In both models, GFP pull‐down assays demonstrated the interaction of TFAP2C with GFP‐PELP1 (Fig. 1A,B). Further, IP with anti‐TFAP2C antibody revealed that TFAP2C interacts with endogenous PELP1 from MCF7 cell lysates, confirming the physiological association between endogenous PELP1 and TFAP2C (Fig. 1C). To further characterize and map the interaction site between TFAP2C and PELP1, we have utilized bacterially expressed full‐length PELP1 and various domains of PELP1 as GST fusion proteins. GST pull‐down assays confirmed the interaction of GST‐PELP1 full length with TFAP2C (Fig. 1D). We then mapped TFAP2C interaction domain using GST fusion of various PELP1 deletions. Ponceau S staining of the gel showed differential mobility of the PELP1‐GST fusion proteins compared with their predicted molecular weights. To illustrate, PELP1‐GST 601–866 aa has a composition that is 25% proline, while PELP1‐GST 1–400 aa has ~ 5% as proline. The presence of higher proportion of proline in PELP1‐GST 601–866 aa contributes to its higher mobility compared with its predicted molecular weight. Western blot analysis of eluates from GST pull‐down assays using PELP1‐deletion fragments showed that the PELP1 region containing 401–600 aa constitutes the major TFAP2C interacting domain (Fig. 1E,F).

### 3.2 PELP1 functions as a coactivator of TFAP2C

**Fig. 2:**
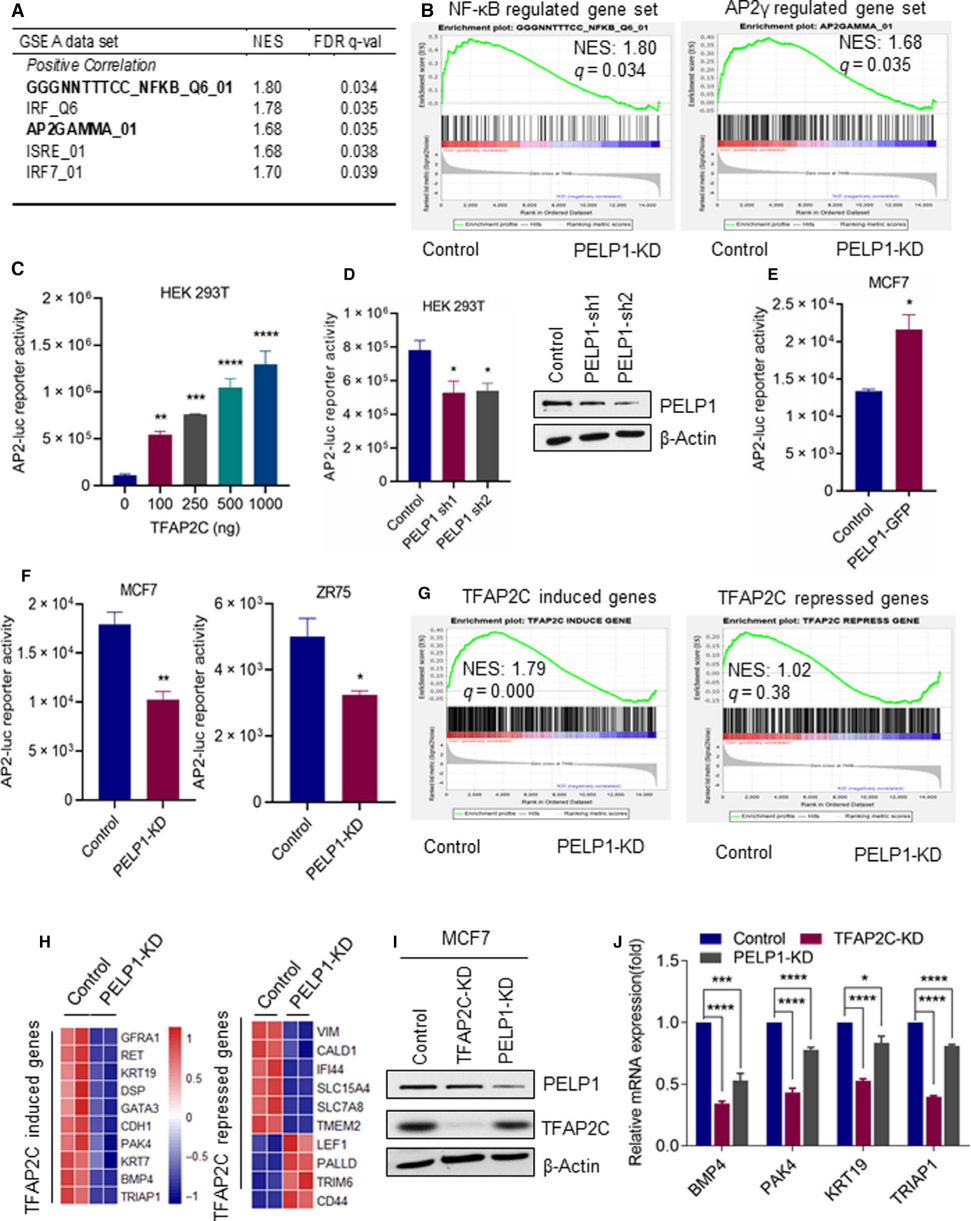
PELP1 serves as coactivator of TFAP2C. (A) Top TFs enriched in PELP1‐regulated genes identified using published PELP1 RNA‐seq data. (B) GSEA of target genes of NF‐κB and TFAP2C with PELP1 RNA‐seq data. (C) HEK293T cells were transfected with AP‐2 Luc plasmid along with increasing concentrations of TFAP2C, and 48h after transfection, the reporter activity was measured. (D) HEK293T cells stably expressing either control or PELP1 shRNA were cotransfected with AP‐2 Luc plasmid along with TFAP2C (100 ng), and 48 h after transfection, the reporter activity was measured. PELP1 knockdown was confirmed using western blotting. (E) MCF7 control and MCF7‐GFP‐PELP1 cells were transfected with AP‐2 Luc plasmid, and the reporter activity was measured after 48 h. (F) MCF7 control and MCF7‐PELP1 shRNA cells or ZR75‐control and ZR75‐PELP1 shRNA cells were transfected with AP‐2 Luc plasmid, and the reporter activity was measured after 48 h. (G) GSEA testing correlation of target genes of PELP1 with genes upregulated by TFAP2C and repressed by TFAP2C. (H) Heat map showing changes in expression levels of several TFAP2C‐upregulated and TFAP2C‐repressed genes in PELP1 RNA‐seq data. (I) TFAP2C knockdown was confirmed using western blotting. (J) Total RNA was isolated from MCF7 model cells expressing either PELP1 shRNA or TFAP2C shRNA, and the status of TFAP2C‐upregulated genes was validated using RT–qPCR. Data are shown as the means ± SEM of three experiments. *P < 0.05; **P < 0.01; ***P < 0.001; ****P < 0.0001 by Student's*t*‐test in C–F and J.

To understand the biological significance of PELP1 interactions with TFAP2C, we examined a previously published PELP1 transcriptome identified by RNA sequencing [14]. We performed GSEA using C3: regulatory target gene sets in the MSigDB database. Results of this analysis revealed that NF‐κB and TFAP2C were the top enriched TFs in the PELP1‐regulated transcriptome (Fig. 2A,B). As a positive control, we confirmed the correlation between PELP1‐ and NFκB‐regulated gene sets, as recently published studies showed that PELP1 functions as a coregulator of NFκB and modulates NF‐κB target genes [27]. Since PELP1 functions as a coregulator of many TFs [8], we examined whether PELP1 functions as coregulator of TFAP2C using AP2‐Luc reporter gene assays. Since HEK293T is a TFAP2C‐negative cell line, we first utilized transient transfection of TFAP2C to establish the functionality of reporter activation using AP2‐Luc reporter [23]. The results from these experiments revealed dose‐dependent activation of the AP2‐Luc reporter by TFAP2C (Fig. 2C). We then repeated these reporter assays, using TFAP2C in HEK293T cells stably expressing PELP1 shRNAs. Downregulation of PELP1 expression using two different PELP1 shRNAs significantly reduced AP2‐Luc reporter activity (Fig. 2D). Further, AP2‐Luc reporter activity was increased when we overexpressed PELP1 in MCF7 cells (Fig. 2E). Moreover, using isogenic BC cells (MCF7, ZR75) with or without PELP1 KD, we found that PELP1 downregulation significantly reduced the AP2‐Luc reporter activity (Fig. 2F). TFAP2C is a TF that is implicated in both activation and repression of genes. To determine whether PELP1 participates in both activation and repression functions of TFAP2C, we conducted GSEA using customized TFAP2C gene sets. TFAP2C‐induced gene set and TFAP2C‐repressed gene set were defined according to TFAP2C microarray data [3] (GSE44203) by *P* value < 0.01 and |log_2_ fold change| > 1. GSEA of TFAP2C‐induced genes showed positive correlation with PELP1; however, no correlation was observed with TFAP2C‐repressed genes (Fig. 2G). We have analyzed expression levels of several TFAP2C‐upregulated and TFAP2C‐repressed genes in PELP1 RNA‐seq data. Many of the known TFAP2C‐induced genes are affected by PELP1 knockdown. Interestingly, PELP1 knockdown did not show a clear trend on many of the TFAP2C‐repressed genes; however, some effect was observed on a few of the TFAP2C‐repressed genes such as CD44 (Fig. 2H). We have confirmed PELP1 regulation of several TFAP2C‐induced genes using RT–qPCR. Western blot analyses confirmed downregulation of TFAP2C protein levels by TFAP2C shRNA (Fig. 2I). RT–qPCR results showed downregulation of TFAP2C‐induced genes in PELP1‐KD cells (Fig. 2J). Collectively, these results suggest that PELP1 and TFAP2C coregulate expression of a subset of TFAP2C genes and further suggest PELP1 may function as coactivator of TFAP2C‐induced genes.

### 3.3 PELP1 knockdown reduced TFAP2C‐mediated clonogenic potential and therapy resistance

**Fig. 3:**
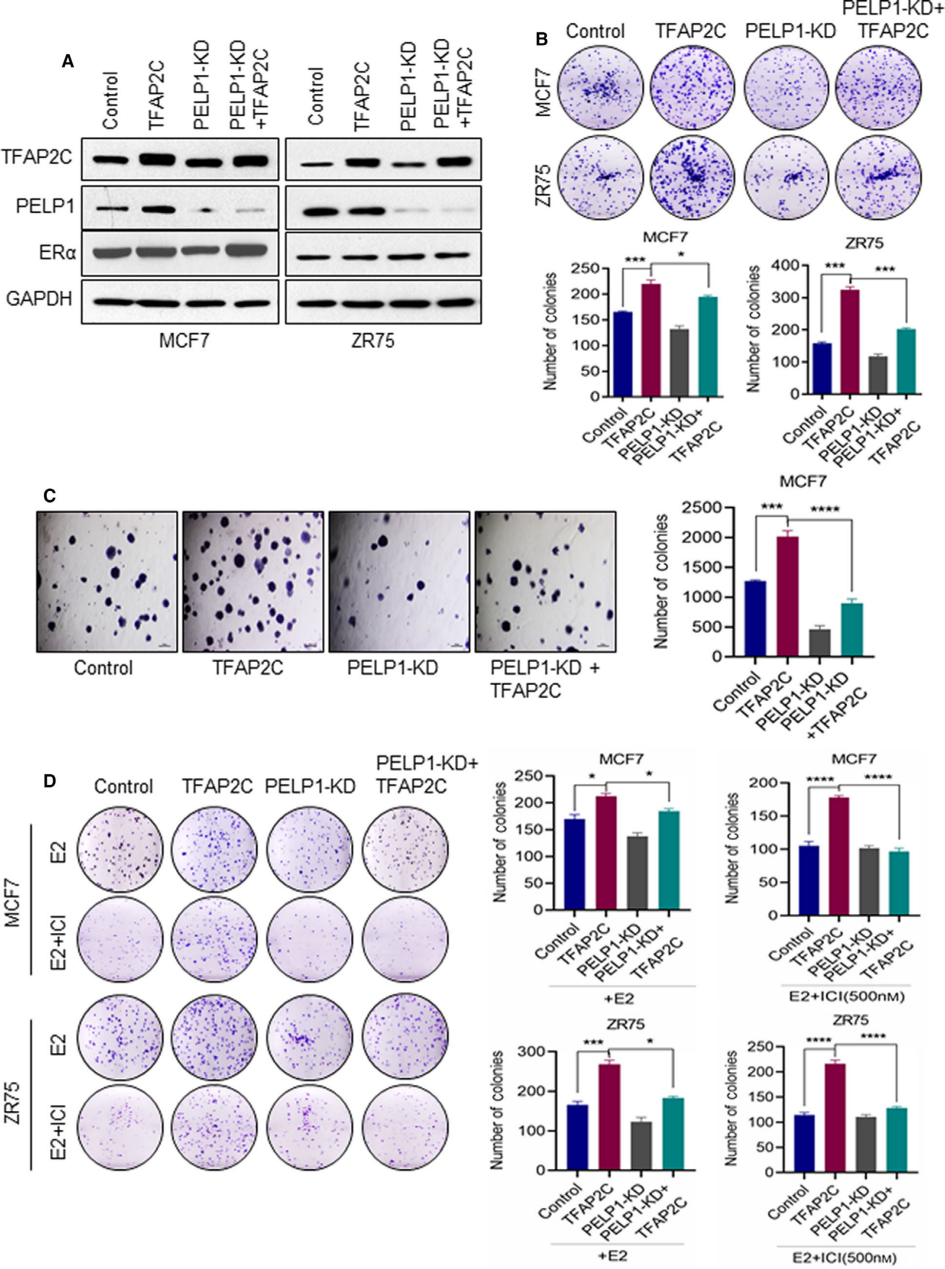
PELP1 is essential for TFAP2C‐mediated oncogenic functions and therapy resistance. (A) Total lysates from MCF7 or ZR75 cells expressing TFAP2C or PELP1 shRNA were analyzed by western blotting for the status of TFAP2C, ERα, and PELP1. Data are shown from two independent experiments. (B) Equal number of MCF7 or ZR75 model cells expressing TFAP2C or/and PELP1 shRNA was plated in six‐well plates and cultured for 14 days, and the number of colonies for each group was counted (*n* = 3). (C) Anchorage‐independent growth potential of the MCF7 cells expressing TFAP2C or/and PELP1 shRNA was measured by soft agar colony formation assay. Representative photographs and quantitation of the soft agar colony formation are shown, scale bar = 100 µm. (D) MCF7 or ZR75 model cells expressing TFAP2C or/and PELP1 shRNA were treated with E2 + Fulvestrant (ICI) for 5 days and then cultured for seven subsequent days. The number of colonies for each group was counted. Data are shown as the means ± SEM of three experiments. *P < 0.05; ***P < 0.001; ****P < 0.0001 by Student's *t*‐test in B–D.

Earlier studies have suggested that TFAP2C plays an important role in the progression of ER+ BC and in the development of resistance to endocrine therapies. To further explore the relationship between PELP1 and TFAP2C in BC and in therapy resistance, we established *in vitro* BC models (MCF7 and ZR75) that overexpress TFAP2C and that underexpress PELP1 (Fig. 3A). As expected from previous studies [2, 3, 4, 5, 6], overexpression of TFAP2C significantly enhanced the colony formation ability of both model cells (Fig. 3B). However, knockdown of PELP1 significantly attenuated TFAP2C‐mediated increase in the colony formation ability of both MCF7 and ZR75 models (Fig. 3B). Furthermore, overexpression of TFAP2C significantly enhanced the soft agar colony‐forming ability of MCF7 cells while knockdown of PELP1 significantly attenuated TFAP2C‐mediated increase in the soft agar colony formation (Fig. 3C). Concomitantly, colony formation assay results demonstrated that TFAP2C overexpression models exhibited resistance to Fulvestrant (ICI) treatment, and TFAP2C‐mediated therapy resistance was significantly decreased when PELP1 expression is knocked down (Fig. 3D).

### 3.4 PELP1 is needed to facilitate optimal histone methyl modifications at the RET promoter

**Fig. 4:**
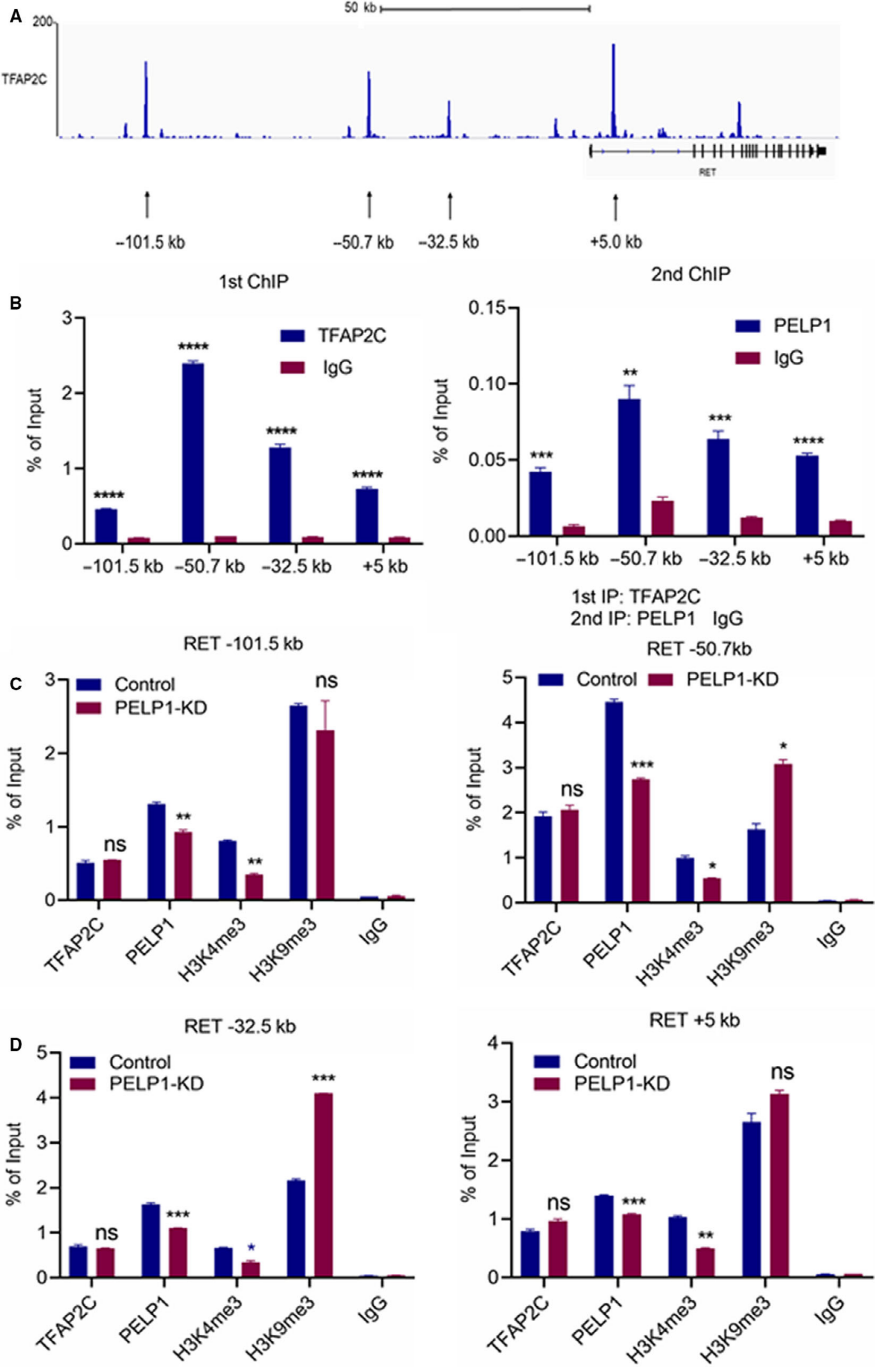
PELP1 is needed to facilitate optimal histone methyl modifications at the RET promoter by TFAP2C. (A) Schematic representation of TFAP2C‐binding sites in the RET promoter based on published TFAP2C ChIP‐seq data in the *RET* locus of MCF7 cells. (B) Primary ChIP was conducted using TFAP2C antibody followed by Re‐ChIP using PELP1 antibody. The status of TFAP2C and PELP1 at the *RET* promoter was determined by qPCR using primers spanning four TFAP2C‐binding sites. (C) Status of the TFAP2C, PELP1, and histone methyl marks H3K4me3 and H3K9me3 in the*RET* promoter was determined by qPCR using primers spanning the four TFAP2C‐binding sites. Isotype‐matched IgG ChIP was used as control. Data are shown as the means ± SEM of two experiments. *P < 0.05; **P < 0.01; ***P < 0.001; ****P < 0.0001 by Student's *t*‐test in B–C.

Previous studies have shown that TFAP2C regulates the expression of proto‐oncogenic RET independent of ER signaling in BC [28]. In addition, TFAP2C‐mediated activation of ERK and AKT via RET is implicated in endocrine therapy resistance. Since our results have indicated that PELP1 functions as a coactivator of TFAP2C‐induced genes; therefore, we examined whether PELP1 plays a role in TFAP2C‐mediated RET expression. PELP1 protein has a histone‐binding domain [12] and facilitates changes in histone methylation via its interactions with histone modifying enzymes [12, 13, 14, 15]. We hypothesized that PELP1 facilitates optimal histone methyl modifications at the RET promoter. To test this, we designed specific primers spanning the TFAP2C‐binding site at the RET gene locus according to a published TFAP2C ChIP‐seq dataset (GSE36351; Fig. 4A). We conducted ChIP and Re‐ChIP experiments to confirm that PELP1 is enriched at the promoter region of the RET gene where TF2APC is recruited. Primary ChIP using TFAP2C antibody followed by Re‐ChIP using the PELP1 antibody confirmed that both PELP1 and TFAP2C are corecruited to the TFAP2C induced promoter (Fig. 4B). Further, results showed that PELP1 knockdown does not affect recruitment of TFAP2C (Fig. 4C,D). We then profiled the status of TFAP2C, PELP1, and histone methyl marks using both H3K9me3 and H3K4me3 antibodies. ChIP results showed that PELP1‐KD decreased enrichment of active histone mark H3K4me3 at −101.5, −50.7, −32.5, and +5.0 kb from the RET transcriptional start site (Fig. 4C,D). On the other hand, PELP1‐KD increased enrichment of inhibitory methyl mark H3K9me3 at −50.7 and −32.5 kb from the RET transcriptional start site (Fig. 4C, D).

### 3.5 TFAP2C‐PELP1 interactions promote activation of RET signaling

**Fig. 5:**
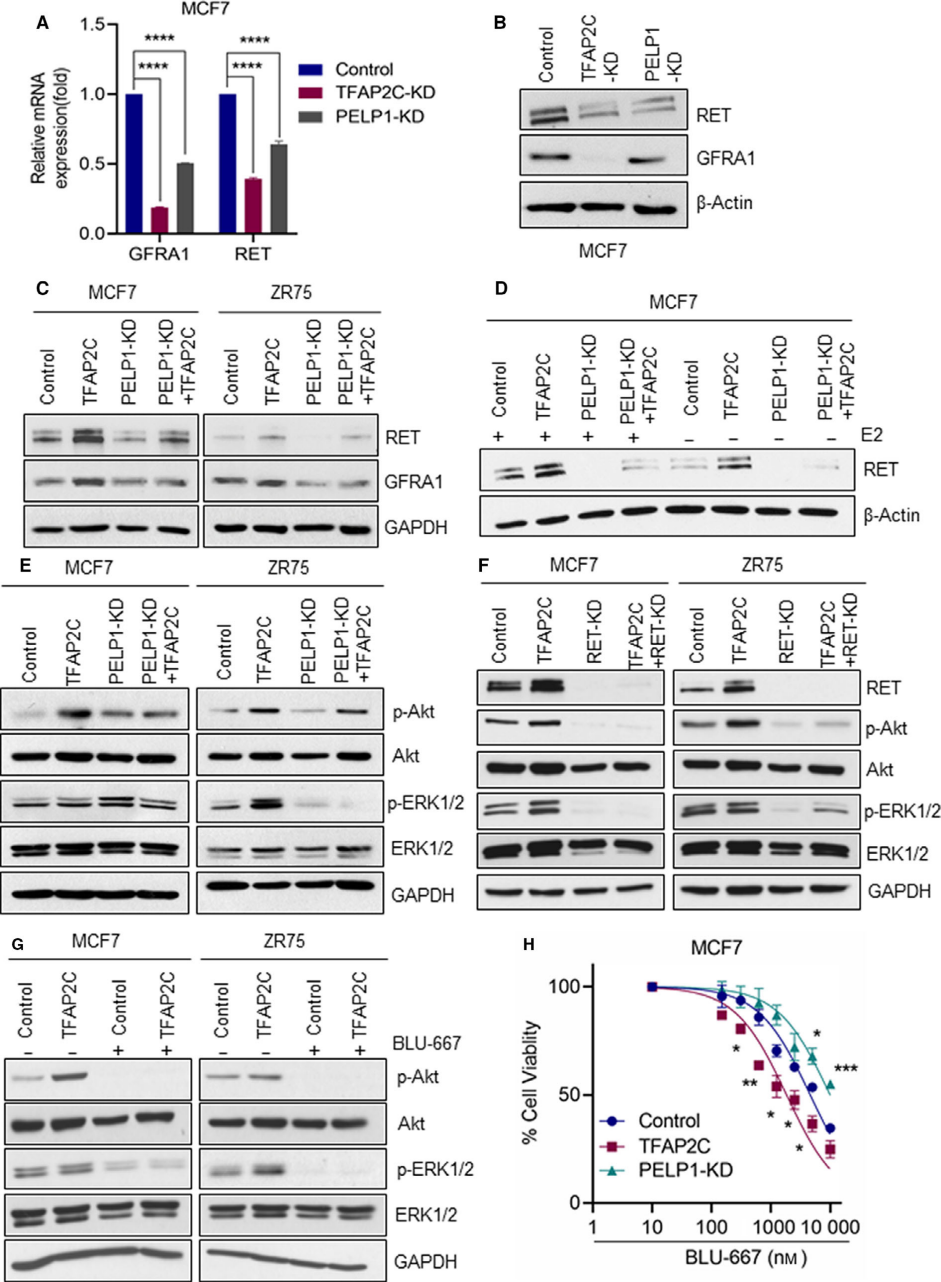
PELP1 is needed for optimal activation of RET signaling by TFAP2C. (A) Total RNA was isolated from MCF7 model cells expressing either PELP1 shRNA or TFAP2C shRNA, and the expression of RET and GFRA1 genes was validated using RT–qPCR. (B) Total lysates from MCF7 cells expressing TFAP2C shRNA or PELP1 shRNA were analyzed by western blotting for the status of RET and GFRA1. (C) Total lysates from MCF7 or ZR75 model cells expressing TFAP2C or/and PELP1 shRNA were analyzed by western blotting for the status of RET and GFRA1. (D) Total lysates from MCF7 or ZR75 model cells expressing TFAP2C or/and PELP1 shRNA were stimulated with E2 for 24h, and the status of RET was analyzed by western blotting. (E) Total lysates from MCF7 or ZR75 model cells expressing TFAP2C or/and PELP1 shRNA were analyzed by western blotting for the status of AKT and MAPK pathways. (F) Total lysates from MCF7 model cells expressing RET‐shRNA were analyzed by western blotting for the status of phospho‐AKT and phospho‐ ERK1/2. (G) Effect of RET inhibitor BLU667 (5 μm) on AKT and ERK1/2 signaling using MCF7 or ZR75 model cells expressing control vector or TFAP2C was measured by western blotting. (H) MCF7 model cells expressing vector or TFAP2C or PELP1 shRNA were plated in 96‐well plates, and cell viability was measured using an MTT assay in the presence or absence of the RET inhibitor BLU667. Data are shown as the means ± SEM of three experiments. *P < 0.05; **P < 0.01; ***P < 0.001; ****P < 0.0001 by Student's *t*‐test in A and H.

We then confirmed whether alterations in histone methylation contribute to increased RET signaling. RT–qPCR and western blot analyses confirmed that RET and its coreceptor GFRA1 are common target genes by TFAP2C and PELP1 (Fig. 5A,B). Western blot analysis of BC model cells overexpressing TFAP2C showed increased expression of RET and its coreceptor GFRA1 (Fig. 5C). In both MCF7 and ZR75 cell models, PELP1‐KD diminished TFAP2C‐mediated elevation in the expression of RET and GFRA1 (Fig. 5C). We repeated these experiments with and without stimulation by E2, and results confirmed E2 regulates RET expression in a TFAP2C‐PELP1‐dependent manner (Fig. 5D). Further, PELP1‐KD also reduced TFAP2C‐mediated activation in AKT and MAPK/ERK pathways (Fig. 5E). We then tested whether RET‐KD also reduced TFAP2C‐mediated activation in AKT and MAPK/ERK pathways. Results confirmed that RET is essential for TFAP2C‐mediated activation of AKT and ERK pathways (Fig. 5F). We further investigated whether RET activation contributes to TFAP2C‐mediated increases in cell proliferation and activation of AKT utilizing a specific RET inhibitor BLU‐667 (Fig. 5G,H). Western blot analyses showed that RET inhibition abolishes AKT and ERK1/2 activation (Fig. 5G). Cell viability assays indicated that TFAP2C overexpression enhances sensitivity of MCF7 cells to RET inhibition, while PELP1 KD cells exhibit resistance to RET inhibition (Fig. 5H). Collectively, these data indicate TFAP2C‐PELP1 interactions pay a key role in promoting RET signaling in BC cells.

### 3.6 TFAP2C‐PELP1 axis promotes tumor growth *in vivo*

**Fig. 6:**
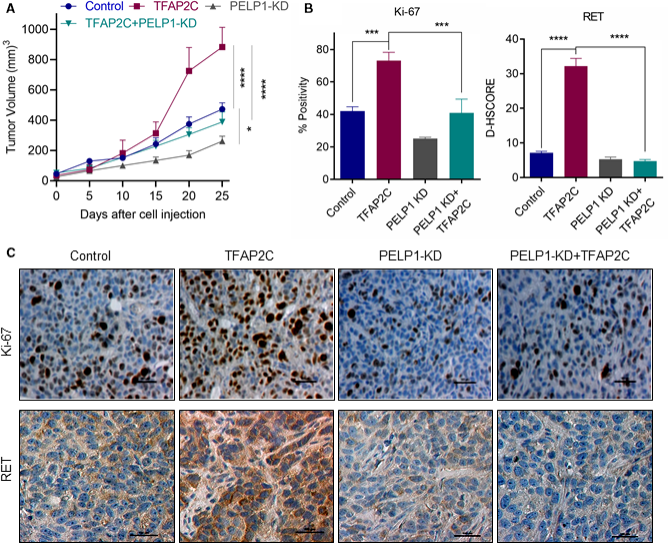
PELP1 is needed for TFAP2C‐mediated BC progression. SCID mice implanted with an E2 pellet were injected with ZR75‐control, ZR75‐TFAP2C, ZR75‐PELP1 shRNA, ZR75‐TFAP2C‐PELP1 shRNA cells into the mammary fat pad, and tumor growth was measured at 5 days intervals. (A) Tumor volume (*n* = 6) is shown in the graph. *P < 0.05; ****P < 0.0001 by two‐way ANOVA with Tukey for *post hoc* test. B,C, Status of Ki‐67 expression as a marker of proliferation and RET was analyzed using IHC. Quantification of Ki‐67 and RET expression is shown (B). (C) Representative IHC images of Ki‐67 and RET are shown. Scale bar = 100µm. Data are shown as the means ± SEM. ***P < 0.001; ****P < 0.0001 by Student's *t*‐test.

We utilized mouse xenografts to examine whether PELP1 is required for TFAP2C‐driven ER+ BC progression *in vivo* using ZR75 BC model cells that express TFAP2C with or without PELP1 KD. ZR75‐TFAP2C xenografts showed increased tumor volume compared with vector transfected cells (Fig. 6A). Further, ZR75‐PELP1 KD + TFAP2C xenografts displayed significantly reduced tumor volumes compared with ZR75‐TFAP2C xenografts. There was no significant difference between ZR75‐PELP1 KD and ZR75‐PELP1 KD + TFAP2C groups. Ki67 staining of the tumor sections revealed greater proliferation in the ZR75‐TFAP2C xenografts than in the ZR75 control and ZR‐75‐PELP1 KD + TFAP2C tumors (Fig. 6B,C). Importantly, ZR75‐PELP1 KD and ZR75‐PELP1 KD + TFAP2C tumors also showed decreased expression of the TFAP2C target gene RET compared with the ZR75‐TFAP2C group (Fig. 6B,C). These results suggest that PELP1 is essential for TFAP2C‐driven tumor growth *in vivo*.

## 4 Discussion

TFAP2C plays an important role in the luminal BC phenotype and oncogenesis [29]; however, the molecular mechanisms by which TFAP2C achieves these functions are not well understood. In this study, we discovered that PELP1 is an essential cofactor for the oncogenic functions exhibited by TFAP2C in ER+ BC. We demonstrated direct interaction between PELP1 and TF2AP2C in BC cell lines and showed that this interaction is necessary for optimal activation of TPAP2C target genes. Further, these studies showed that PELP1 plays a role in the activation of oncogenic RET signaling via modifying histone methylation status at the TFAP2C‐binding site. Finally, we showed that PELP1 plays a critical role in TPAP2C‐mediated tumor progression *in vivo*. Collectively, these findings implicate PELP1 as a novel coregulator of TFAP2C and the PELP1‐TFAP2C axis plays a role in BC progression and development of therapy resistance.

TFAP2C participates in ER transcriptional regulation [30]. Here, we identified PELP1 as another essential cofactor needed for optimal TFAP2C functions. PELP1 is a scaffold protein that functions as a coregulator of several NRs including ER [8] but can also function as coregulator of other TFs such as E2F [31]. Notably, the PELP1 protein has a histone‐binding domain [12], recognizes histone modifications, and interacts with several chromatin‐modifying complexes [12, 13, 14, 15]. RNA‐seq studies using an ER+ BC cell line identified 318 genes as PELP1‐regulated genes, many of which are involved in BC progression [14, 15]. The results from this study suggested that some of the PELP1‐regulated genes contain the TFAP2C motif in the promoter region. Importantly, knockdown of PELP1 significantly downregulated expression of the genes that contained a TFAP2C motif in the promoter. Our results suggest that PELP1 is only involved in the regulation of TFAP2C upregulated genes but not in downregulated genes. Furthermore, reporter gene assays confirmed that PELP1 functions as a coregulator of TFAP2C. ChIP and Re‐ChIP experiments further confirmed that both PELP1 and TFAP2C are corecruited to TFAP2C‐induced promoters. Since PELP1 functions as a scaffold protein that interacts with multiple chromatin modulating proteins, the location of TFAP2C‐repressed genes and chromatin structure may play a role in the differential regulation. We acknowledge that future studies are clearly needed using global ChIP for further understanding of the differential regulation of TFAP2C target genes by PELP1.

Increased TFAP2C expression contributes to the failure of BC cells to exhibit growth arrest following endocrine therapy contributing to sustained growth and poorer patient outcome [32]. In addition, high transcript and protein levels of TFAP2C correlated well with decreased response to Fulvestrant treatment [33]. Furthermore, TFAP2C regulation of BC cells involves both ER‐dependent and ER‐independent activation of RET receptor pathways [28]. RET expression is a negative prognostic indicator in BC and plays an important role in endocrine therapy resistance [34, 35]. Our results suggest that PELP1 indeed plays a critical role in the TFAP2C‐mediated regulation of RET and its coreceptor GFRA1 expression. Furthermore, PELP1‐mediated epigenetic changes appear to play a critical role in the TFAP2C regulation of RET expression.

PELP1 expression is deregulated in breast tumors, and PELP1 protein expression is an independent prognostic predictor of shorter BC‐specific survival. Elevated expression is positively associated with markers of poor outcome [16]. In addition, its dysregulation contributes to BC therapy resistance [17, 18]. Similarly, higher TFAP2C expression correlates with poor overall survival after a 10 year of diagnosis of ERα+ and endocrine therapy‐treated subgroups [36]. Our data suggests that TFAP2C and PELP1 signaling plays a role in ER+ BC progression and that the PELP1 dysregulation, commonly seen in breast tumors, may contribute to enhanced TFAP2C oncogenic signaling. Dysregulation of both TFAP2C and PELP1 in ER+ BC suggests that modulation of the TFAP2C pathway by PELP1 may represent a potential mechanism by which PELP1 promotes breast tumorigenesis. Further, our results suggest that PELP1 is essential in TFAP2C‐mediated endocrine therapy resistance as PELP1 KD reduced TFAP2C‐mediated resistance to endocrine therapies.

PELP1 plays an essential role in several signaling pathways including ER signaling [9, 10, 11]. Previous studies have shown that overexpression of PELP1 in the mammary glands of transgenic mouse models contributes to mammary gland carcinoma, further supporting its oncogenic potential *in vivo* [37]. Concomitantly, treatment of BC xenografts with PELP1‐siRNA liposomes significantly reduced tumor volume [18]. TFAP2C signaling is implicated in malignant progression of many cancers [38, 39, 40]. Specific to our research, TFAP2C is implicated in mammary oncogenesis [41] and is commonly amplified in BC [42]. It is known that PELP1 functions as a proto‐oncogene; therefore, we hypothesized that PELP1 interactions with TFAP2C may increase TFAP2C tumorigenic potential. In agreement with this hypothesis, our data demonstrate that PELP1 knockdown reduced TFAP2C‐mediated ER+ BC progression *in vivo*.

## 5 Conclusions

In summary, our data provides the first evidence to the best of our knowledge that PELP1 functions as a coregulator of TFAP2C. We also provide evidence to indicate that PELP1 is essential for optimal TFAP2C‐mediated transcriptional functions and BC progression. Based on these findings, we predict that TFAP2C interactions with PELP1 confer a growth advantage to BC cells by activating an oncogenic RET signaling cascade thus contributing to BC progression and therapy resistance.

## Conflict of interest

The authors declare no conflict of interest.

## Author contributions

JL, GRS, HZ, SV, and RKV designed the experiments and interpreted the results. JL, ZL, YL, ML, UPP, WT, SV, KAA, YZ, and XL conducted the experiments; YL and SV conducted Xenograft studies; JL, KAA, HZ, GRS and RKV wrote the manuscript.
