# Corrigendum to: Interaction of transcription factor AP‐2 gamma with proto‐oncogene PELP1 promotes tumorigenesis by enhancing RET signaling

**DOI:** 10.1002/1878-0261.13129

**Published:** 2021-11-10

The authors of [1] would like to add a third address under the affiliations of Ratna K Vadlamudi:

Audie L. Murphy Division, South Texas Veterans Health Care System, San Antonio, TX 78229, USA.
